# The Direct Superior Approach in Total Hip Arthroplasty: A Systematic Review

**DOI:** 10.2106/JBJS.RVW.23.00182

**Published:** 2024-03-15

**Authors:** Bart van Dooren, Rinne M. Peters, Alies M. van der Wal-Oost, Martin Stevens, Paul C. Jutte, Wierd P. Zijlstra

**Affiliations:** 1 Department of Orthopedics, University of Groningen, University Medical Center Groningen, Groningen, the Netherlands; 2 Department of Orthopedics, Medical Center Leeuwarden, Leeuwarden, the Netherlands; 3 Department of Orthopedics, Martini Hospital, Groningen, the Netherlands; 4 Kennisinstituut van Medische Specialisten, Federatie Medisch Specialisten, Utrecht, the Netherlands

## Abstract

**Background:**

Evolution of the surgical approach for total hip arthroplasty (THA) has led to the development of the minimally invasive direct superior approach (DSA). It is hypothesized that the DSA reduces postoperative pain and hospital length of stay (LOS). We aimed to provide an overview of current evidence on clinical, functional, and radiological outcomes with respect to risk of revision, complications, pain scores, physical function, operative time, LOS, blood loss, radiological outcomes, and learning curve.

**Methods:**

A comprehensive search of Medline, Embase, Web of Science, Cochrane Central Register of Controlled Trials, and Google Scholar, reported according to the Preferred Reporting Items for Systematic Reviews and Meta-Analyses literature search extension guidelines, was conducted to identify studies evaluating clinical, functional, and radiological outcomes of the DSA. Quality assessment was performed using the Cochrane Risk of Bias tool and Newcastle-Ottawa Scale. The review protocol was prospectively registered in the International Prospective Registry of Systematic Reviews.

**Results:**

Seventeen studies were included, generally of moderate quality. Qualitative synthesis evidenced accurate implant positioning, short LOS, and a short learning curve. Conflicting findings were reported for postoperative complications compared with conventional approaches. Better functional outcomes were seen in the early postoperative period than the posterolateral approach (PLA). Outcomes such as blood loss and operative time exhibited conflicting results and considerable heterogeneity.

**Conclusion:**

Based on moderate-certainty evidence, it is uncertain if the DSA provides short-term advantages over conventional approaches such as PLA. There is limited evidence on long-term outcomes post-THA using the DSA. Further studies and ongoing registry monitoring is crucial for continuous evaluation of its long-term outcomes.

**Level of Evidence:**

Level III. See Instructions for Authors for a complete description of levels of evidence.

The selection of the surgical approach for total hip arthroplasty (THA) in daily practice is determined by surgeon experience, training, and personal preferences^1^. The posterolateral approach (PLA) is the most frequently used technique for THA, although a shift to the direct anterior approach (DAA) is observed more recently^2^. The DAA is known to have a reduced revision risk for dislocation, early mobilization, and reduced hospital length of stay (LOS) compared with the PLA, but a higher risk of femoral-sided revision and a steep learning curve is reported^3-7^.

To decrease dislocation rates and improve the early recovery of patients undergoing the traditional PLA, the direct superior approach (DSA) was introduced. The DSA, a modification of the PLA, preserve the iliotibial band and short external rotators, excluding the piriformis and conjoint tendon^8,9^. It is hypothesized that the DSA may help reduce postoperative pain, intraoperative blood loss, and hospital LOS^10-13^. By contrast, it is hypothesized that minimally invasive THA may lead to increased risk of complications such as component malposition and femoral stem undersizing^14^. Finally, implementation of new surgical approaches is associated with a learning curve in which higher operative times and complication rates can be encountered^5-7^. Although numerous studies suggest a benefit for patients undergoing minimally invasive THA, some surgeons remain skeptical that these changes in surgical technique are responsible for the observed improvements^15^.

In this systematic review, we aim to provide an overview of current evidence on clinical, functional, and radiological outcomes in primary THA performed using the DSA. Complication rates, reasons for revision, pain scores, physical function, operative time, hospital LOS, blood loss, radiological outcomes, and learning curve were explored.

## Methods

### Protocol and Registration

A systematic review was conducted according to the Preferred Reporting Items for Systematic Reviews and Meta-Analyses guidelines^16^. The protocol of this systematic review was prospectively registered in the International Prospective Registry of Systematic Reviews (ID: CRD42022371913).

### Surgical Technique

The objective of the DSA is to preserve the iliotibial tract, obturator externus tendon, and quadratis femoris muscle^17^. The procedure involves a small incision along the posterior edge of the greater trochanter, extending in the proximal direction. An incision is made through the gluteus maximus fibers, skin, subcutis, and gluteus maximus fascia, ensuring preservation of the iliotibial band. The gluteus maximus is incised. The conjoined tendon and piriformis tendon are detached, marked, and positioned posteriorly. Lifting the gluteus minimus, a capsulotomy is performed in line with the femoral neck. Subsequently, the hip is dislocated, and this is succeeded by the resection of the femoral neck, reaming of the acetabulum, and the insertion of the femoral and acetabular components. Long DSA Hohmann retractors and specialized reamers are used for this procedure. The capsule is closed side to side; the piriformis reattached; and the fascia, subcutaneous tissue, and skin closed in layers. Detailed illustrations of the DSA are displayed in a comprehensive article by Barrett et al.^9^.

### Eligibility Criteria

Studies were eligible if (1) the authors reported on the outcome of primary THA through the DSA; (2) at least 10 adult patients were included; (3) full text was available; (4) operative technique was defined; and (5) written in English, French, Dutch, or German. Exclusion criteria were (1) cadaveric studies, (2) no original research, (3) no full text available, (4) former systematic reviews, (5) animal studies, (6) hip hemiarthroplasties, and (7) revision procedures. Furthermore, we excluded alternative posterior-oriented minimally invasive methods, including the supercapsular percutaneously assisted total hip, due to its specific emphasis on preserving the piriformis muscles and avoiding intraoperative hip dislocation.

### Search Strategies, Information Sources, and Study Selection

Studies investigating the outcome of the DSA to THA were identified using Cochrane Central Register of Controlled Trials (CENTRAL), Medline, Embase, Web of Science, and Google Scholar on December 18, 2023. The Medline search strategy was developed and transferred into similar search strategies for the other databases in collaboration with a clinical librarian (A.M.v.d.W.-O.). The search in all databases was performed with a combination of the following keywords: “arthroplasty,” “hip replacement,” “hip prosthesis,” “direct superior,” “direct superior approach,” “iliotibial,” and “transpiriformis” (Table S1 supplementary data). References of the included articles were screened to identify additional studies. Eligibility assessment was performed by 2 independent reviewers (B.v.D. and R.M.P.) using Rayyan^18^. Disagreements were solved by consulting the senior author (W.P.Z.).

### Data Extraction

Data extraction was performed independently by 2 authors (B.v.D. and R.M.P.). Any disagreement was resolved by discussion between the reviewers. In case of no consensus, the conflict was resolved by the senior author (W.P.Z.). The following data were extracted: study design, study population, author, publication year, country, patient characteristics (age, gender, body mass index [BMI]), operative treatment strategy, length of follow-up, and outcome. Primary outcome measures were (1) revision rates, (2) type of complication, (3) pain and physical function measured with patient-reported outcome measures, and (4) radiological outcomes as assessed using plain pelvic and hip radiographs. Secondary outcome measures were (1) operative time, (2) LOS, (3) blood loss, and (4) learning curve.

### Quality Appraisal and Risk of Bias Assessment

Two authors (B.v.D. and R.M.P.) independently assessed the risk of bias and methodological quality of the included studies. Randomized controlled trials (RCTs) were assessed using the Cochrane Risk of Bias tool. A study is judged to have high risk of bias if at least 1 domain scores as such. Observational studies were assessed with the Newcastle-Ottawa Scale, which consists of 8 items with 3 subscales and a maximum score of 9 for these 3 subscales. The quality of the studies was determined based on the obtained scores: low quality 0 to 3, moderate quality 4 to 7, and high quality 8 to 9.

### Data Synthesis and Analysis

Owing to the small number of studies, the retrospective design, large heterogeneity of the control groups, and differences in outcomes reported, we were not able to perform a quantitative synthesis of the data as it would be methodologically unsound. Therefore, data are described narratively in the Results section. A summary of findings for each outcome in which 3 or more studies are selected for this review is provided.

## Results

### Study Selection

**Fig. 1:**
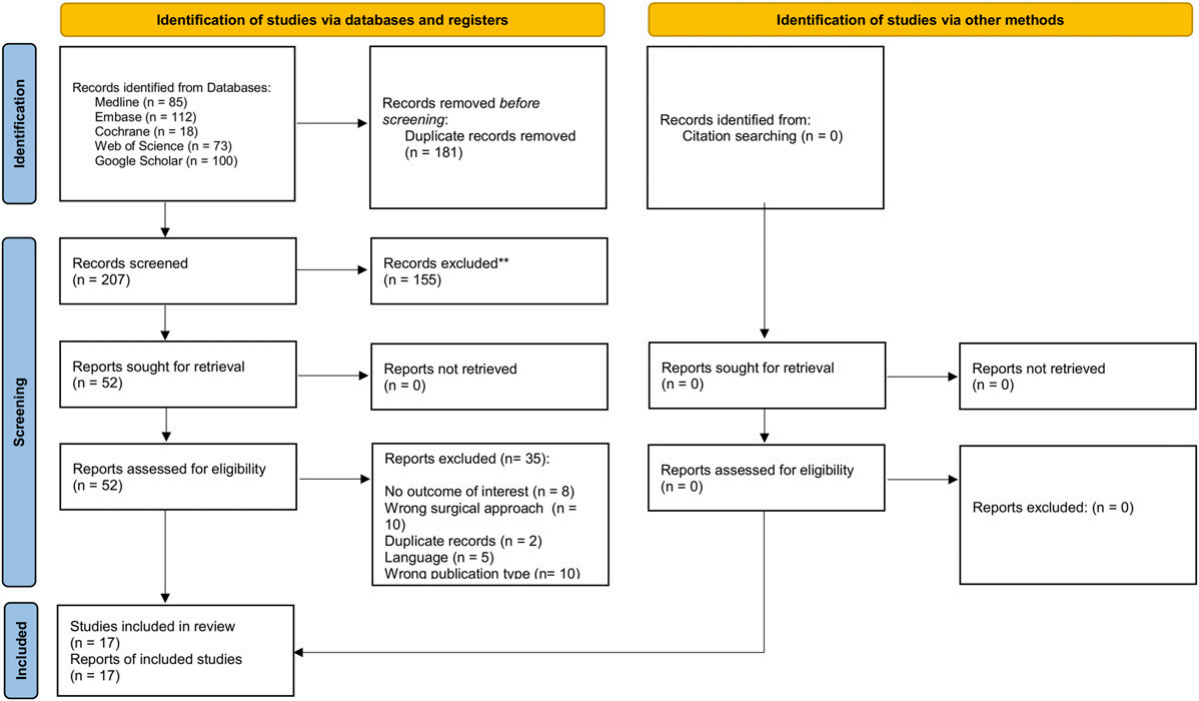
PRISMA flow diagram of included studies. PRISMA = Preferred Reporting Items for Systematic Reviews and Meta-Analyses.

We identified 388 studies from electronic databases searches. One hundred eighty-one studies were excluded after reviewing duplications using EndNote, and 207 articles were screened by title and abstract, of which 52 articles were eligible for full-text screening. Ultimately, 17 qualifying articles were selected^10-13,17,19-30^ (Fig. 1).

### Quality Assessment and Methodology

**Fig. 2:**
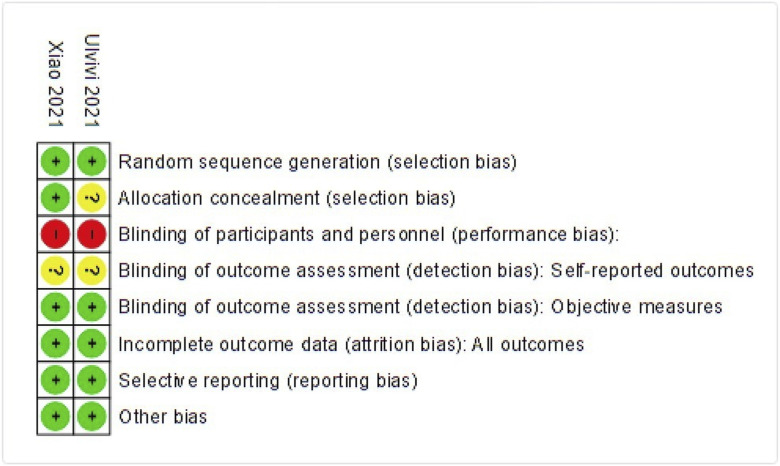
Risk of bias summary using the Cochrane Risk of Bias tool for randomized trials. Low (+), high (−), or unclear (?) risk of bias.

The characteristics of included studies are presented in Table I. Seventeen studies including 3,551 patients met the inclusion criteria^10-13,17,19-30^. The number of patients per study ranged from 20 to 1,341, with a mean age range of 51 to 74 years (Table I). Follow-up ranged from 3 months to 2.7 years. Fifteen studies had an observational design, including 2 register studies (n = 1,341 and n = 343)^29,30^. Two studies were RCTs^26,28^. We found that most studies had a moderate level of evidence (Tables II and III; Fig. 2). The major methodological limitations were retrospective design, small sample sizes, and no independent blind assessment of end points.

**TABLE I:** Characteristics of the Included Studies*

Author, Year Country	Study Design	Follow-up (SD)	No. of Patients DSA (n)	Control Group (n)	Age, yr Mean (Range)	Sex (Male/Female), %	BMI (kg/m^2^)	Outcome Variables†
Roger and Hill, 2012^17^ United States	Retrospective cohort study (case series)	22 (14-33) mo	135	n.a.	72 (45-92)	51/49	27.3 (19.5-40)	2-3, 8-11, 14
Duijnisveld et al., 2020^19^ Netherlands	Prospective cohort study	12 mo	52	MPA‡ 52	DSA: 69 ± 8.4 MPA: 69 ± 8.4	46/54	25 ± 3.4	2-11, 13
Ezzibdeh et al., 2021^10^ United States	Retrospective case-controlled study	2.7 ± 0.7 yr	20	MPA‡ 20	DSA: 51 ± 12 MPA: 64 ± 9	50/50	26 ± 5	2-3, 8-12
Ezzibdeh et al., 2020^20^ United States	Retrospective cohort study (case series)	108 ± 36 d	301	n.a.	63 ± 10	43/57	28.4 ± 5.3	2, 8-10, 12
Ezzibdeh et al., 2020^21^ United States	Retrospective cohort study (case series)	108 ± 28 d	40 (20 vs. 20)	MPA‡ 20	1st 20 DSA: 58 ± 10 2nd 20 DSA: 51 ± 12 MPA: 64 ± 9	65/35 vs. 50/50	28 ± 5	3, 8-13
Kenanidis et al., 2022^12^ Greece	Retrospective case-controlled study	12 mo	100	PL 100	DSA: 65.4 ± 8.4 PL: 65.5 ± 7.9	42/58	28.38 ± 3.09	1-3, 4, 6, 8-11, 14
Korth et al., 2021^22^ United States	Retrospective case series	2.2 ± 0.4 yr	40	n.a.	55 ± 12	57.5/42.5	27 ± 5	2-3, 8-12, 14
Leonard and Ohly, 2021^13^ UK	Retrospective case-controlled study	1 yr	100	PL 100	DSA: 68.0 (26-88) PL: 68.05 (29-89)	61/39	28 (16.9-50.4)	2, 4, 5, 8-11, 13
LeRoy et al., 2020^11^ United States	Retrospective cohort study	1 yr	403	PL 273	DSA: 63.4 ± 10.4 PL: 63.4 ± 9.2	50.5/49.5 vs. 47.1/52.9	29.5 ± 6 vs. 28.1 ± 5.1	2, 8-10
Nam et al., 2016^23^ USA United States	Prospective cohort study	Minimum 1 yr 2.8 ± 1.0 yr	42	MPA‡ 196	DSA: 63.9 ± 6.1 MPA: 52.4 ± 6.1	—	—	4
Siljander et al., 2020^24^ United States	Retrospective case-controlled study	3 mo	333	DAA 1.846 PL 3.162	DSA: 62 ± 11 DAA: 65 ± 10 PL: 64 ± 11	46/54	27.4 ± 4.4	1, 2, 8, 10
Tsiridis et al., 2020^25^ Greece	Prospective cohort study	1 yr	200	n.a.	66.53 ± 8.87 (49-87)	35.5/64.5	27.59 ± 2.98	2, 3, 6, 8-11, 14
Ulivi et al., 2021^26^ Italy	Prospective RCT	6 mo	22	PL 23	DSA: 74 ± 8.9 PL: 72 ± 7.7	32/68	23 ± 2.8	2-4, 6, 8-10, 12
Watanabe et al., 2021^27^ Japan	Retrospective case-controlled study	2 ± 0.4 yr	30	PL 30	DSA: 68.7 ± 8.8 PL: 66.2 ± 9.4	17/83	24 ± 3.7	2, 11
Xiao et al., 2021^28^ China	Prospective RCT	3 mo	49	PL 57	DSA: 71.06 ± 10.87 PL: 73.93 ± 10.02	33/67	27.73 ± 4.18	3, 4, 8, 14
van Dooren et al., 2023^29^ Netherlands	Retrospective cohort study	1.6 yr	1,341	DAA 56.626 PL 117.576	DSA: 68.21 ± 10.1 PLA: 69.1 ± 10.6	37/63	26.4 ± 4.1	1, 2
van Dooren et al., 2023^30^ Netherlands	Retrospective cohort study	1 yr	343	DAA 15.017 PL 22.616	DSA: 68.0 ± 8.5 DAA: 68.6 ± 9.1 PLA: 69.1 ± 9.1	35/65	26.1 ± 4.0	4, 5, 6, 7

* BMI = body mass index, DAA = direct anterior approach, DSA = direct superior approach, EQ5D = EuroQol-5 Dimension, HOOS-PS = Hip Disability and Osteoarthritis Outcome Score-Physical Function Short Form, LOS = length of stay, MPA = mini posterior approach, NRS = Numerical Rating Scale, OHS = Oxford Hip Score, PL = posterolateral, RCT = randomized controlled trial, and VAS = visual analog scale.

† 1, risk of revision; 2, complications; 3, Harris Hip Score; 4, pain scores (NRS VAS); 5, OHS; 6, HOOS-PS; 7, EQ5D; 8, operative time; 9, blood loss; 10, time to hospital discharge LOS; 11, accuracy of implant position; 12, gait analysis; 13, learning curve; 14, incision length.

‡ Not available, due to the categorized nature of data.

**TABLE II:** Risk of Bias Assessment Using the Newcastle-Ottawa Quality Assessment Scale for Cohort Studies*

Cohort Study									
Studies	Selection				Comparability	Outcome			Total Quality
Author, Year	Representativeness of the Exposed Cohort	Selection of the Nonexposed Cohort	Ascertainment of Exposure to Implants	Demonstration of the Absence of Outcome of Interest at the Start of the Study	Comparability of Cohorts Based on Design or Analysis	Assessment of Outcome	Was Follow-up Long Enough to Have Outcomes	Adequacy of Follow-up of Cohorts	
Roger and Hill, 2012^17^	★	☆	★	★	☆☆	☆	★	☆	4/9 moderate
Duijnisveld et al., 2020^19^	★	★	★	★	★☆	☆	★	★	7/9 moderate
Ezzibdeh et al., 2020^21^	★	★	★	★	☆☆	☆	★	☆	5/9 moderate
Ezzibdeh et al., 2020^20^	★	★	★	★	☆☆	☆	★	☆	5/9 moderate
Korth et al., 2021^22^	★	☆	★	★	☆☆	☆	★	★	5/9 moderate
LeRoy et al., 2020^11^	★	★	★	★	★☆	★	★	☆	7/9 moderate
Nam et al., 2016^23^	★	★	★	★	☆☆	☆	★	☆	5/9 moderate
Siljander et al., 2020^24^	★	★	★	★	★★	★	★	☆	8/9 high
Tsiridis et al., 2020^25^	★	☆	★	★	★☆	★	★	★	7/9 moderate
van Dooren et al., 2023^29^	★	★	★	★	★★	★	★	★	9/9 high
van Dooren et al., 2023^30^	★	★	★	★	★★	★	★	☆	8/9 high

* A study can be given a maximum of 1 star for each numbered item within the Selection and Outcome categories. A maximum of 2 stars can be given for Comparability. A star was left white when it was not allocated. The quality of the studies was determined on the basis of the obtained scores: low quality 0 to 3, moderate quality 4 to 7, and high quality 8 to 9.

**TABLE III:** Risk of Bias Assessment Using the Newcastle-Ottawa Quality Assessment Scale for Case-Control Studies*

Case Control									
Studies	Selection				Comparability	Exposure			Total Quality Score
Author, Year	Is the Case Definition Adequate?	Representativeness of the Cases	Selection of Controls	Definition of Controls	Comparability of Cases + Controls	Ascertainment of Exposure	Same Ascertainment Method for Cases + Controls	Non-Response Rate	
Ezzibdeh et al., 2021^10^	☆	★	★	★	☆☆	☆	★	☆	4/9 moderate
Kenanidis et al., 2022^12^	★	★	★	★	★☆	★	★	★	8/9 high
Leonard and Ohly, 2021^13^	☆	★	★	★	☆☆	☆	★	☆	4/9 moderate
Watanabe et al., 2021^27^	★	★	★	★	★☆	☆	★	☆	6/9 moderate

* A study can be given a maximum of 1 star for each numbered item within the Selection and Exposure categories. A maximum of 2 stars can be given for Comparability. A star was left white when it was not allocated. The quality of the studies was determined on the basis of the obtained scores: low quality 0 to 3, moderate quality 4 to 7, and high quality 8 to 9.

### Outcomes

#### Complications

Fourteen studies reported on complication rates (Table IV). Complication rates were reported at different time points (3 months to 2.7 years). Overall, 51 complications (1.6%) were reported, and in 39 cases (1.3%), revision surgery was needed. The most common reason for revision was periprosthetic fracture. Eight cases (0.3%) of postoperative dislocation were observed, for which 5 revisions were performed. No revisions for cup malpositioning were reported. One revision due to excessive leg lengthening and 1 revision for inadequate offset were reported^17^.

**TABLE IV:** Number of Complications for the DSA per Study*

	Roger and Hill, 2012^17^ n = 135	Duijnisveld et al., 2020^19^ n = 52	Ezzibdeh et al., 2021^10^ n = 20	Ezzibdeh et al., 2020^20^ n = 301	Ezzibdeh et al., 2020^21^ n = 40	Kenanidis et al., 2022^12^ n = 100	Korth et al., 2021^22^ n = 40	Leonard and Ohly, 2021^13^ n = 100	LeRoy et al., 2020^11^ n = 403	Siljander et al., 2020^24^ n = 333	Tsiridis et al., 2020^25^ n = 200	Ulivi et al., 2021^26^ n = 25	Watanabe et al., 2021^27^ n = 30	van Dooren et al., 2023^29^ n = 1,341	Total N = 3,117
Mean follow-up	22 mo	12 mo	2.7 yr	108 d	90 d	12 mo	2.2 yr	1 yr	2 yr	3 mo	1 yr	6 mo	2 yr	1.6 yr	
Surgical complication rate, n (%)	3 (2.2)	2 (3.8)	0 (0)	7 (2.3)	0 (0)	1 (1)	0 (0)	1 (1)	0 (0)	10 (3)	2 (1)	3 (12)	1 (3.3)	21 (1.6)	51 (1.6)
Revision rate, n (%)	2 (1.5)	2 (3.8)	0 (0)	4 (1.3)	0 (0)	1 (1)	0 (0)	1 (1)	0	5 (1.5)	2 (1)	1 (4)	0 (0)	21 (1.6)	39 rev; 1.3 and 8 (0.5) treated intraoperatively
Complication type															
Dislocations	0	0	0	0	0	0	0	N/R	0	2† (1 r)	0	2†	0	4	8 (5; 0.2% rev)
Superficial wound infection	0	0	0	N/R	0	1	0	N/R	0	N/R	1	0	0	N/R	2 (2; 0.1% rev)
Deep wound infection	0	0	0	N/R	0	0	0	N/R	0	1 (1 r)	1	0	0	4	6 (6; 0.2% rev)
Intraoperative fracture	1‡	0	0	3	0	0	0	0	0	3‡	0	0	1	N/R	8 (8; 0.3% rev)
Postoperative fracture	N/R	2	0	4	0	N/R	0	1	0	4 (3 r)	N/R	1	0	4	16 (15; 0.5% rev)
Loosening	0	N/R	0	N/R	0	N/R	0	N/R	0	N/R	N/R	0	N/R	7	7
Sciatic nerve palsies	0	0	N/R	N/R	0	0	N/R	N/R	N/R	N/R	0	0	0	N/R	0
Thromboembolic event	0	0	N/R	2	N/R	0	N/R	N/R	N/R	2	0	0	N/R	N/R	4
Other§	2 (2 r)	0	0	0	0	0	0	0	0	0	0	0	0	2	6 (4; 0.1% rev)

* DSA = direct superior approach, N/R = not reported, OSM = oncostatin M, r = revision(s), and rev = revised.

† Treated with closed reduction

‡ OSM during primary surgery.

§ Defined as excessive leg lengthening and/or inadequate offset.

Seven studies compared DSA with PLA complication rates^11-13,24,26,27,29^. Ulivi et al. performed an RCT comparing the postoperative results of 25 DSA patients with 25 PLA patients. They reported a higher rate of adverse events (2 dislocations and 1 periprosthetic fracture) for the DSA (3/25; 12%) compared with 1 (thromboembolic event) for the PLA (1/25; 4%). Both dislocations were treated with closed reduction^26^. One high-quality large register study reported a lower overall risk of revision (1.6% vs. 3.1%) and dislocation (0.3% vs. 1.0%) for the DSA (n = 1,341) compared with the PLA (n = 117,576)^29^. In addition, no difference in risk of revision for periprosthetic fracture was found (0.3% vs. 0.4%). By contrast, another high-quality retrospective observational statewide registry study found a significantly higher early revision rate (p = 0.015) for the DSA (n = 333 with 1.5% revisions) compared with the PLA (n = 3,162 with 0.4% revisions)^24^. The difference in revision rates in the latter study was due to the number of periprosthetic fractures. By contrast, no difference in dislocations or infection was reported. Four other observational studies did not find any difference in complication rates between the DSA and the PLA or mini posterior approach (MPA)^11-13,27^. Overall, while a RCT with a small sample size suggest a disadvantage for the DSA, larger real-world evidence studies present different outcomes.

#### Pain

Seven studies reported on postoperative pain scores^12,13,19,23,26,28^. Four studies^12,13,19,28^ reported on immediate postoperative visual analog scale scores. Xiao et al. found lower pain scores for the DSA than for the standard PLA in the first 3 postoperative days (p < 0.001)^28^. By contrast, 1 high-quality study found no significant difference in pain scores between the DSA and the PLA on the first postoperative day and last day of hospitalization^12^. We found through moderate to high-quality studies that postoperative pain levels did not differ clinically between DSA and PLA patients in the first 5 days or after 1, 2, 3, and 4 weeks; 3 months; and 6 months^13,26,30^.

Duijnisveld et al. reported comparable pain scores between the DSA and MPA on the first postoperative day and last day of hospitalization and no difference in pain scores during rest and activity at 3-month and 1-year follow-ups^19^. Finally, 1 study with moderate evidence reported no difference in the incidence of moderate-to-severe pain between the DSA and MPA over the trochanter, anterior thigh, or lateral thigh at a minimum of 1-year follow-up^23^.

#### Physical Function

The most commonly used instrument to measure postoperative recovery was the Harris Hip Score (HHS), where higher scores represent better physical functioning. In total, 9 studies reported on functional scores after using the HHS^10,12,17,19,21,22,25,26,28^. Mean HHSs in the early postoperative phase were better for the DSA than for the PLA in 1 high-quality study and 1 RCT^12,28^ (Table V): Both studies reported significantly higher HHS for the DSA than for the PLA after 1-month follow-up. One RCT found significantly higher HHS for the DSA than for the PLA at 1-week follow-up^26^. By contrast, another RCT reported no difference in functional improvement (HHS) after 1-week and 1-month follow-ups^26^. None of the included studies found a significant difference in HHS between the DSA compared with their PLA or MPA counterparts at 3-month, 6-month, 1-year, or 2-year follow-up^10,12,22,25,26^.

**TABLE V:** Preoperative and Postoperative HHSs*

Study†	Groups	HHS Baseline	HHS 1 mo	HHS 3 mo	HHS 6 mo	HHS 1 yr	HHS 2 yr	Outcome
Korth et al., 2021^22^	DSA	57 (17)		88 (12)		96 (6)	97 (5)	Significant HHS improvement (p < 0.001) compared with baseline. Plateau by 2 yr (p = 0.359)
Tsiridis et al., 2020^25^	DSA	44.8 (5)	80 (4.6)	87.9 (5)		91.4 (5.4)		Significant HHS improvement (p < 0.001) at 1, 3, and 12 mo compared with baseline.
DSA vs. MPA								
Duinisveld 2020^19^	DSA	56 (16)		87 (16.9)		87 (16.2)		Significant HHS improvement (p < 0.001) compared with baseline. No difference between DSA and MPA (p = 0.75).
	MPA	54 (15)		85 (16.4)		89 (14.8)		
Ezzibdeh et al., 2021^10^	DSA	56 (11)		89 (12)		N/R	98 (6)	Significant HHS improvement (p < 0.001) compared with baseline. No difference between DSA and MPA at 3 mo (p = 0.72) and 2 yr (p = 0.389).
	MPA	61 (20)		89 (10)		N/R	96 (11)	
Ezzibdeh et al., 2020^21^	DSA	56 (11)		89 (11)				No difference in mean HHS between DSA and MPA at 3 mo (p > 0.05)
	MPA	61 (20)		89 (10)				
DSA vs. PL approach								
Kenanidis et al., 2022^12^‡	DSA	44.5 (3.6)	81.6 (3.3)	88.9 (3.3)	92.6 (3.6)			Significant HHS improvement at all follow-up times (p < 0.001) compared with baseline. Greater functional improvement for the DSA at 1 mo (P < 0.001) compared with the PLA.
	PL	44.4 (3.8)	77.8 (4.0)	88 (3.7)	92.7 (2.3)			
Ulivi et al., 2021^26^§	DSA	N/R	N/R	N/R	N/R			Significant HHS improvement (p < 0.001) at 6 mo compared with baseline. No statistically significant differences between groups. Plateau after 3 mo.
	PL	N/R	N/R	N/R	N/R			
Xiao et al., 2021^28^‡§	DSA	N/R	N/R	N/R				Significant HHS improvement from baseline to 1 wk, 1 mo, and 3 mo. DSA had higher HHS at 1 wk and 1 mo (p = 0.00).
	PL	N/R	N/R	N/R				

* DSA = direct superior approach, HHS = Harris Hip Score, MPA = mini posterior approach, N/R = not reported, and PLA = posterolateral approach.

† Roger and Hill (2012) not reported due to unknown time point of HHS measurement; N/R, numbers not reported.

‡ Significant difference between groups.

§ Numbers missing since the results were reported in figures

Four moderate-to-high–evidence studies reported patient-reported outcomes using the Hip Disability and Osteoarthritis Outcome Score (HOOS) questionnaire^12,25,26^, a hip-specific questionnaire intended to evaluate symptoms and functional limitations of patients suffering from hip dysfunction. One RCT reported no difference in HOOS between the DSA and the PLA after 1 week, 2 weeks, 3 weeks, 1 month, 3 months, and 6 months^26^. Second, a high-quality study reported significantly higher functional scores for the DSA at 1-month follow-up than for the PLA. After 1 month, DSA and PLA functional scores were comparable^12,26,30^.

### Radiological Outcomes

Nine studies reported on acetabular cup inclination angle^10,13,17,19-22,25,27^, and 7 studies reported on acetabular cup anteversion^10,13,17,21,22,25,27^ (Table VI). Most studies use the Lewinnek safe zone (5-25° anteversion, 30-50° inclination) to measure optimal implant positioning^31^. Five studies reported a mean cup inclination and mean cup anteversion within the Lewinnek safe zone^10,20-22,27^. The DSA showed cup inclination and anteversion comparable with the MPA and PLA^10,27^. Ezzibdeh et al. found that the mean cup anteversion was slightly higher for the MPA (16 ± 4°) than for the DSA (12 ± 3°)^21^. Leonard et al. reported a significantly higher cup inclination and lower cup anteversion for the DSA than for the PLA^13^. Still, both studies reported that all THAs were within the Lewinnek safe zone.

**TABLE VI:** Radiological Parameters*

Study	Method	Groups	Cup Inclination	Cup Anteversion	Implant Positioning
Roger and Hill, 2012^17^	AP radiograph	DSA	41 (21-49)	21 (15-27)	No revisions for component malpositioning
Korth et al., 2021^22^	AP radiograph	DSA	38.8 (5.4)	12.9 (3.7)	All DSAs within the Lewinnek safe zone
Tsiridis et al., 2020^25^	AP radiograph	DSA	44.15 (33.5)	20.76 (3.59)	No revisions for component malpositioning
Ezzibdeh et al., 2020^20^	AP radiograph	DSA	42 ± 5	N/R	All DSAs within the Lewinnek safe zone
DSA vs. MPA					
Duijnisveld et al., 2020^19^	AP radiograph	DSA	51 (6)	N/R	Acetabular inclination higher for the DSA compared with the MPA (p = 0.028)
		MPA	54 (7)	N/R	
Ezzibdeh et al., 2021^10^	AP radiograph	DSA	39.1 (5.4)	13.5 (4.4)	All DSAs within the Lewinnek safe zone
		MPA	38.3 (5.8)	16 (4.4)	
Ezzibdeh et al., 2020^21^	AP radiograph	DSA	1st 20 cases: 39 (6) 2nd 20 cases: 39 (5)	1st 20 cases: 12 (3) 2nd 20 cases: 13 (4)	MPA slightly more anteversion compared with first 20 DSA cases (p = 0.006). All cases within the Lewinnek safe zone.
		MPA	38 (6)	16 (4)	
DSA vs. PLA					
Leonard and Ohly, 2021^13^	AP radiograph	DSA	46.3 (6.1)	13.9 (6.8)	Statistically different inclination and anteversion between DSA and PLA
		PL	42.7 (5.8)	18.5 (6.9)	
Watanabe et al., 2021^27^	CT image	DSA	42.1 (5.3)	21.9 (6.9)	All DSA within the Lewinnek safe zone
		PL	41.8 (6.5)	22.3 (11.7)	

* AP = anteroposterior, CT = computed tomography, DSA = direct superior approach, MPA = mini posterior approach, and PLA = posterolateral approach.

#### Hospital LOS (Days)

LOS was recorded in 11 studies as a measure of recovery. In the majority of studies, the LOS of patients undergoing a DSA to THA was significantly reduced compared with that of PLA or MPA patients^10-13,21,24^. Four studies compared DSA with PLA LOS, all reporting a significantly lower LOS for the DSA than for the PLA^11-13,24^. Two studies with moderate evidence reported a significantly lower LOS for the DSA than for the MPA^10,21^. Duijnisveld et al. reported no difference in LOS between the DSA and MPA^19^.

#### Operative Time

Twelve studies have investigated operative time. Considerable heterogeneity in mean operative time was seen (range 57-130 minutes). Six studies compared DSA with PLA mean operative times^11-13,24,26,28^: 3 moderate-to-high–evidence studies found a shorter operative time for the DSA than for the PLA^11,24,28^, 1 study reported longer operative time for the DSA (90 ± 14 minutes) than for the PLA (77 ± 20 minutes)^26^, and 2 studies reported no difference in operative time^12,13^. Duijnisveld et al. reported significantly longer operative time for the DSA than for the MPA^19^. Ezzibdeh et al. reported comparable operative time for the first 20 DSA THAs than for the first 20 MPA THAs, yet a significant decrease in operative time was seen for the second 20 DSA THAs, suggesting a learning curve effect^21^.

#### Blood Loss

Blood loss in ml was recorded in 10 studies^10,12,13,17,19-22,25,26^. Three studies compared DSA with PLA blood loss^12,13,26^. Ulivi et al. showed significantly lower BL for the DSA than for the PLA (149 vs. 225 mL, respectively, p = 0.04)^26^. Other studies reported no statistically significant differences in DSA and PLA blood loss (data not shown). Three studies compared DSA with MPA blood loss^10,19,21^. Duijnisveld reported comparable BL between the DSA and MPA^23^ while Ezzibdeh et al. reported lower blood loss for the DSA than for the MPA^10,21^.

#### Incision Length

A total of 5 studies with 524 procedures reported on incision length in centimeters. The mean incision length varied from 8.9 (SD = 2.3) to 9.16 (SD = 1.25) cm. Two studies reported statistically significantly shorter incision lengths for the DSA than for the PLA^12,26^.

#### Learning Curve

Three studies reported on the learning curve^13,19,21^. First, Ezzibdeh et al. reported that the learning curve for the DSA is lower in 20 patients^21^. The authors reported similar intraoperative blood loss and LOS when comparing the first 20 DSA cases with the second set of DSA THAs. Duijnisveld et al. reported that the DSA was found to have no learning curve in terms of implant positioning^19^. No significant differences were found in the DSA group for operative time, blood loss, or change in perioperative hemoglobin level, suggesting the absence of a learning curve. Finally, Leonard et al. reported no difference in operative time and estimated mean blood loss between the first 100 DSA THAs and a matched PLA cohort^13^. All DSA cases, including the one with a learning curve, were included in their study.

## Discussion

The DSA is suggested as a minimally invasive surgical approach for THA, aiming to optimize outcome for THA patients. The DSA may provide an earlier functional recovery with slightly better functional scores in the first postoperative month. However, after 3 months, no differences in functional scores were seen compared with the PLA and MPA. The DSA enables adequate implant positioning and resulted in a shorter LOS compared with the PLA. Finally, the learning curve for the DSA seems to be short. However, no differences were reported in pain scores. Finally, most studies show no significant differences in complication rates, but 1 RCT reported higher complications with DSA, in contrast to another large registry study. Hence, we found through moderate-certainty evidence that the DSA may not offer advantages over alternative approaches in terms of complication rates and pain scores.

Currently, there is 1 narrative review from Kayani et al. and 1 review and meta-analysis from Zang et al.^8,32^ with findings similar to ours. Higher quality studies with bigger numbers have been published since. Our study, building on previous work by Kayani et al. and Zang et al., extends the literature on DSA outcomes. In comparison with the systematic review by Zang et al., our study takes a more comprehensive approach, incorporating a broader range of outcomes such as complications, pain scores, radiological results, operative time, and learning curve. In addition, our study includes a larger number of studies, particularly incorporating those from 2023, as well as integrating data from 2 large registry studies^24,29,30^.

Perhaps the most important outcome with a new surgical approach is the complication risk associated with it, particularly in the initial stages of the learning curve. The DSA seems to be associated with low risk of postoperative dislocations, sciatic nerve palsies, loosening, and wound complications. Although the short-term complication risk seems to be low for the DSA, the evidence presented in the study does not strongly support the conclusion that the DSA offers significant benefits over conventional approaches in terms of complication rates. The most common reason for short-term revision for the DSA was intraoperative and postoperative fractures. Most notably, intraoperative fractures have been related with difficulty in exposing and manipulating the femur during femoral preparation. Based on current literature, the rate of intraoperative femoral fractures ranges from 0% to 5.3%, regardless of surgical approach^33-36^. Recently, Bruggeman et al. conducted a large registry study using the Norwegian Arthroplasty Register including 218,423 primary THAs, reporting an intraoperative periprosthetic fracture rate of 1.0%^36^. In our review, we found an intraoperative periprosthetic fracture rate of 0.3% and overall periprosthetic fracture rate of 0.8% for the DSA, which is consistent with current literature.

Second, we found that the DSA may provide an earlier functional recovery with slightly better functional scores in the first postoperative month. This early functional recovery could be explained by the muscle-sparing and iliotibial band-preserving nature of the DSA. However, it has been difficult to objectively determine precisely which factors are responsible for improved patient outcomes. We cannot rule out that some surgeons may have selectively applied the DSA to nonobese and younger patients who are predisposed to recover quickly. However, most studies reporting differences in functional outcomes between the DSA and control-matched patients based on sex, age, and BMI or reported no difference in sex, age, or age between groups. However, it is important to note that some studies with comparable functional scores between the DSA and controls were based on low-level evidence, and the patient demographics may not have been adequately matched in all included studies. Therefore, the exact benefits of the DSA in terms of early recovery remain unclear.

Third, the importance of implant positioning and its impact on short-term and long-term outcomes is well established in THA. Poor implant positioning can result in increased dislocation rates, component impingement, increased surface wear, and reduction of implant survivorship^37^. In DSA THAs, acetabular bone resection is performed using specialized reamers. Achieving adequate implant position can be more challenging. In this study, we found that DSA THAs can be performed safely in terms of implant positioning. Direct access to the acetabulum and femur seems easily obtained. The majority of studies reported a mean cup inclination and mean cup anteversion within the Lewinnek safe zone. None of the included studies reported revisions for cup malpositioning or femoral stem undersizing. One revision due to excessive leg lengthening and 1 revision for inadequate offset was reported^17^. Hence, the DSA seems to be safe in terms of implant positioning.

Finally, we found considerable heterogeneity in surgical outcomes such as operative time and blood loss. In our opinion, the heterogeneity in outcomes might be explained by the nature of the included studies in terms of their local protocols, surgeon experience, enhanced recovery pathways, use of modern anesthetic techniques, and the routine use of tranexamic acid. Most studies did not consider confounding factors when comparing blood loss; therefore, it was difficult to draw conclusions about blood loss in the DSA compared with other approaches. We think that operative time is an outcome parameter that is difficult to generalize since it can be influenced by multiple confounding factors (e.g., surgeon experience, adequate exposure, assistance, or operation room team). Moreover, during the surgeon's learning curve, there usually is a prolonged operative time. The wide variation in operative times for the DSA supports the idea that the surgeon's proficiency plays an important role and shows that there is potential for a shorter operative time.

This review has some limitations. First, most included studies were retrospective cohort studies and case series, so the level of evidence is less robust and more prone to selection, reporting, and interpretation bias. Second, we were not able to pool data owing to the small number of studies, the retrospective design, the large heterogeneity of the control groups, and differences in outcomes reported. Hence, we did not proceed to a quantitative synthesis. Third, most studies were conducted by a single surgeon specifically trained in the posterior approach and DSA to THA. Thus, surgeon experience and annual case volume may vary between the included studies. Moreover, the evidence in this systematic review does not strongly support significant benefits of the DSA over alternative methods. A limitation includes the need for careful patient education, clarifying that “superior” refers to an anatomical direction, not superiority in outcomes. Discussions with patients should focus on individual factors, preferences, and potential benefits or risks associated with different approaches. Finally, due to insufficient data, important outcome parameters such as postoperative gait analysis could not be considered. Our findings should be interpreted with caution given these limitations.

## Conclusion

Based on presently available moderate-certainty evidence, it is uncertain if the DSA provides short-term advantages over conventional approaches such as PLA. However, registry data indicate lower revision rates for dislocation with the DSA, offering valuable real-world insights. There is currently a lack of evidence on its long-term efficacy and safety. Further prospective studies are needed, and ongoing registry monitoring is crucial for continuous evaluation of its long-term outcomes.

### Sources of Funding

No benefits in any form have been received or will be received related directly or indirectly to the subject of this article.

## Appendix

Supporting material provided by the authors is posted with the online version of this article as a data supplement at jbjs.org (http://links.lww.com/JBJSREV/B79). This content was not copyedited or verified by JBJS.
